# Experimental Study on the Stability and Distribution of Air Voids in Fresh Fly Ash Concrete

**DOI:** 10.3390/ma15238332

**Published:** 2022-11-23

**Authors:** Yanhai Wang, Hang Lu, Rui Xiao, Wei Hu, Baoshan Huang

**Affiliations:** 1 Department of Civil and Environmental Engineering, The University of Tennessee, Knoxville, TN 37996, USA; 2 Louisiana Transportation Research Center, Louisiana State University, Baton Rouge, LA 70808, USA; 3 Biotechnology, Engineering, and Skilled Technologies Division, Wake Technical Community College, Raleigh, NC 27603, USA

**Keywords:** concrete, fly ash, air-entraining admixture, loss on ignition, air voids

## Abstract

The air void system purposely introduced by an air-entraining admixture (AEA) is of great significance for the protection of concrete from freeze–thaw damage. Fly ash has been globally used in concrete, while the unburnt carbon in fly ash can adsorb AEA molecules and, thus, increase the AEA demand. Previous studies primarily focused on the air content of fresh fly ash concrete. This paper aimed to explore the stability and distribution of air voids in fly ash concrete at the fresh state. To achieve this goal, eleven different fresh fly ash concrete mixtures with an initial air content of 6 ± 1% were prepared in the laboratory. Samples were taken at various times within 75 min after initial mixing to investigate the air content and air void distribution in fly ash concrete at the fresh state using a super air meter (SAM). The results indicated that there was no significant correlation between loss on ignition (LOI) of fly ash and AEA demand to achieve the initial air content of 6 ± 1%. Class C fly ash concrete tended to have a better air content retention than Class F fly ash concrete. Compared with LOI, AEA demand had a stronger correlation with air content retention. Most of the fly ash concrete mixtures had a satisfactory air void system immediately after mixing, but the SAM number showed an increasing trend over time, suggesting the coarsening of the air void system with time.

## 1. Introduction

Concrete is an artificial construction material mainly comprising cementitious materials, aggregate, water, and chemical admixtures in appropriate proportions. Concrete has been widely used in various buildings and infrastructure worldwide. However, as a typical porous media material, concrete is usually prone to deterioration under harsh environmental conditions. For example, due to water invasion, concrete is susceptible to freeze–thaw damage in cold regions. To reduce the freeze–thaw deterioration, the most commonly adopted method in the world is to use air-entrained concrete according to the theoretical hypothesis that the purposely introduced air voids can provide extra space to decrease expansion pressure induced by freezing water [1,2].

Various test methods have been employed to determine the air void system of concrete. For fresh concrete, some common tests mainly include the ASTM C231 [3] pressure method, ASTM C138 [4] gravimetric method, and ASTM C173 [5] volumetric method. Among these methods, the pressure method is the most commonly used in the concrete industry for quality control/assurance due to its simplicity and convenience on the jobsite. Although the above test methods have many advantages, some limitations also affect their effectiveness. For instance, the measured air content of fresh concrete cannot characterize the spacing of air voids, which is more critical to improving the freeze–thaw resistance [6]. It has been widely reported that although some fresh concrete met the air content requirement specified by agencies, the freeze–thaw test as per ASTM C666 [7] indicated that the corresponding hardened concrete was not freeze–thaw-durable [8,9]. For hardened concrete, the parameters of the air void distribution such as spacing factor can be measured based on ASTM C457 [10], which is a tedious and time-consuming test. In addition, much faster and more convenient test methods such as image processing [11,12,13], ultrasonic scattering measurement [14,15], X-ray CT [6,16], 2D to 3D unfolding techniques [17], macro-photography [18], and the photometric stereo method [19,20] have been developed to obtain the air void distribution in hardened concrete. Nonetheless, as these tests are conducted on hardened concrete, the results obtained are significantly behind the construction schedule. Therefore, it is crucial to determine the air void distribution in concrete at the fresh state.

To address this need, in the early 1990s, the air void analyzer (AVA) was invented to obtain the distribution of air voids in fresh concrete based on Stoke’s law [21]. The AVA test method has been standardized under AASHTO TP 75 [22]. However, the AVA test device is relatively expensive. In addition, this test is very sensitive to environmental conditions, which is not suitable for application in the field. Due to these challenges, the AVA has rarely been used by the concrete industry [23].

More recently, a new test device named the super air meter (SAM) was invented to obtain the air void distribution in fresh concrete [24]. The SAM is a modified version of the traditional ASTM C231 Type B pressure meter, which involves applying three successive pressures of 14.5, 30, and 45 psi in two sequences to obtain the air content and SAM number. The SAM test procedure has been issued by AASHTO TP 118 [25]. Compared with the AVA, the SAM is not expensive and is easily performed in the field. More importantly, both laboratory and field studies have proved that SAM number had a good correlation with the spacing factor and freeze–thaw durability factor [26,27,28,29,30], indicating that the SAM is a useful tool to ensure concrete structures have enough freeze–thaw resistance. For example, a spacing factor of 0.2 mm or below was recommended by the American Concrete Institute to ensure that concrete structures have adequate resistance against freeze–thaw damage [31], while a SAM number of 0.2 or below can be utilized to predict a spacing factor of 0.2 mm or below with 88% accuracy [26,27].

Fly ash, a byproduct of coal ignition, has been globally used in concrete over many years considering economic, environmental, and technological benefits. For example, adding fly ash to concrete can reduce the carbon footprint of cement production, and decrease the cement hydration heat in early ages and, thus, the thermal-cracking probability [32]. Furthermore, fly ash addition can enhance the later-age mechanical and durability performance of concrete because of the pozzolanic reaction [33]. The fly ash content in concrete is usually moderate, accounting for 15% to 30% of cementitious material by mass [34]. According to ASTM C618 [35], fly ash can be divided into two types: one is Class C or high-calcium fly ash, which typically originates from subbituminous or lignite coal; the other is Class F or low-calcium fly ash, which usually originates from bituminous or anthracite coal. However, partially replacing cement with fly ash had an adverse influence on the production of air voids in concrete: the air content of fly ash concrete was lower than cement concrete at the same air-entraining admixture (AEA) dosage [36,37,38,39,40]. The main reason is that fly ash usually has a low fraction of unburnt carbon particles, while the non-polar carbon surfaces provide active adsorption sites for AEA molecules, causing a decreased amount of AEA for stabilizing the entrained air voids and, thus, a reduced air content [41]. To compensate for the air content loss, an increased AEA dosage is often needed for fly ash concrete. On the other hand, ASTM C618 [35] specifies a maximum loss on ignition (LOI) of 6% for the fly ash used for concrete.

The alkali content in cement usually affects the air void stability in concrete. For example, Dubovoy et al. [42] reported that the concrete containing cement with a low-alkali content (0.21%) needed twice the AEA dosage than concrete containing cement with a moderate or high-alkali content (around 0.60% or greater) to obtain the same air content. In addition, the concrete containing cement with a low-alkali content had a more unstable air void system, i.e., the spacing of air voids increased from 0.15 mm to 0.53 mm during the 80 min interval. Plante et al. [43] suspected that a high alkali content in the cement helped to produce stable air voids in fresh concrete. Pigeon et al. [44] validated that the increase in soluble alkali content in cement improved the air void stability in fresh concrete.

However, previous studies mainly investigated the air content of fresh fly ash concrete, while the air void stability and distribution have seldom been reported. To this end, this paper aimed to explore the stability and distribution of air voids in fresh fly ash concrete. Eleven types of fly ash from different sources meeting ASTM C618 [35] were used to replace 20% cement by mass to produce fresh fly ash concrete mixtures in the laboratory, and a neutralized vinsol-resin AEA was used to obtain an initial air content of 6 ± 1%. Samples were taken at 0, 25, 50, and 75 min after the initial mixing of concrete mixtures; then, the air content and air void distribution were obtained by the SAM according to AASHTO TP 118 [25]. 

## 2. Materials and Methods

### 2.1. Materials

A Type I cement meeting ASTM C150 [45] and eleven different types of fly ash (five Class C, FA1~FA5; six Class F, FA6~FA11) conforming to ASTM C618 [35] were utilized as the cementitious materials. The chemical and physical characteristics of cement are presented in Table 1. The alkali content in cement (Na_2_O + 0.658 K_2_O) of this study was 0.53% based on Table 1, which could basically be considered a moderate level. The 1-, 3-, 7-, and 28-day compressive strength of cement was 15.4, 24.9, 31.2, and 39.6 MPa, respectively. Table 2 shows the classification and physical properties of fly ash. Three tests by composition were carried out per test and the average was reported. Crushed limestone aggregate and natural river sand conforming to ASTM C33 [46] were utilized as the coarse and fine aggregate, respectively. The limestone aggregate had a nominal maximum size of 19 mm. The river sand was siliceous sand. The fineness modulus of natural river sand was 2.67. The moisture absorption of limestone aggregate and natural river sand was 0.83% and 1.15%, respectively. The specific gravity of limestone aggregate was 2.74 and the specific gravity of natural river sand was 2.62. The gradation of coarse and fine aggregate is listed in Table 3. A commercially available polycarboxylate superplasticizer (SP) meeting ASTM C494 [47] was utilized to improve the workability of fresh concrete. A commercially available neutralized vinsol-resin air-entraining admixture (AEA) meeting ASTM C260 [48] was used for air entrainment.

### 2.2. Mix Proportions

A total of eleven fly ash concrete mixtures were produced in the laboratory. Table 4 presents the mix proportions of these mixtures. For each mixture, the fly ash content in the cementitious materials was 20% by mass. The mass ratio of water to cementitious materials (w/cm) was kept constant at 0.4. Considering that the fly ash content in cementitious materials was relatively low (20% by mass) and the different types of fly ash in this study had similar specific gravity (varied from 2.48 to 2.75), all the eleven fly ash concrete mixtures had the same concrete proportion, as shown in Table 4. Other researchers have also adopted the same approach to studying the air content of concrete containing different fly ash [38,39]. Trial mixing was performed and the dosage of SP and AEA was determined to get a slump of 150 ± 25 mm and an initial air content of 6 ± 1% immediately after mixing.

### 2.3. Test Methods

Concrete ingredients including cement, fly ash, coarse and fine aggregate, and tap water with SP and AEA were mixed in an 85 L drum mixer as per ASTM C192 [49] in the laboratory room at the ambient temperature of 22 ± 2 °C. The coarse and fine aggregates acquired from a local ready-mix concrete plant were oven-dried to obtain a constant moisture content between batches, and extra water was used to make up for the water absorption of aggregates. Before mixing, all raw materials were placed in the laboratory for at least 1 day to make sure that they were preconditioned to the same temperature.

**Figure 1:**
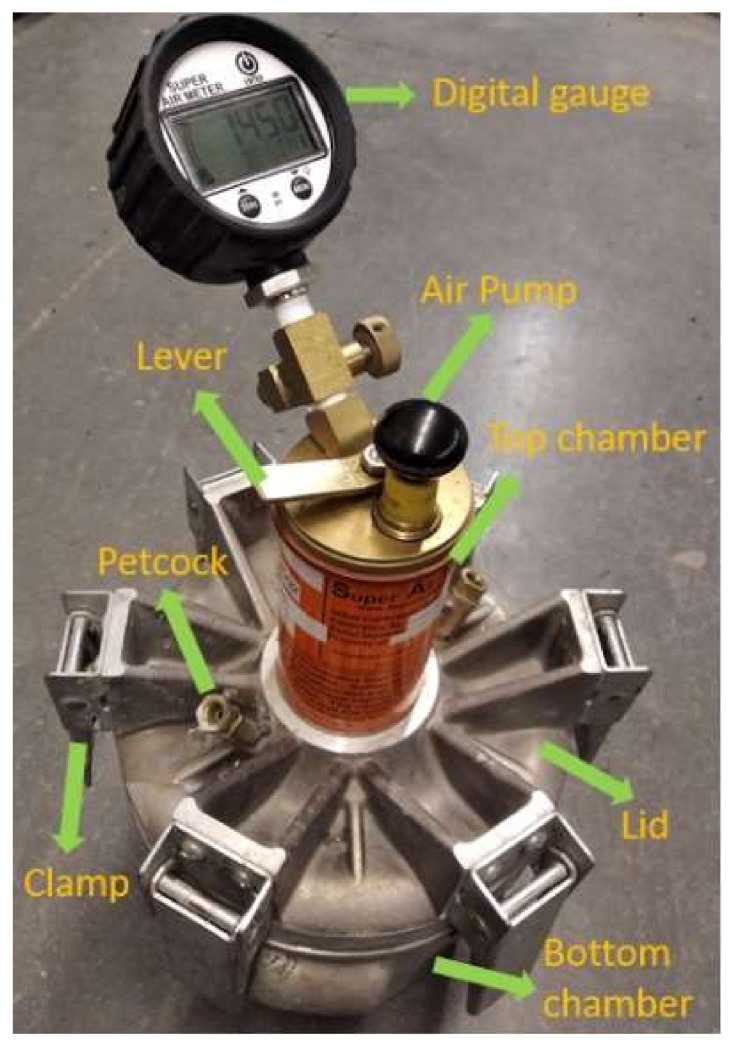
SAM equipment.

**Figure 2:**
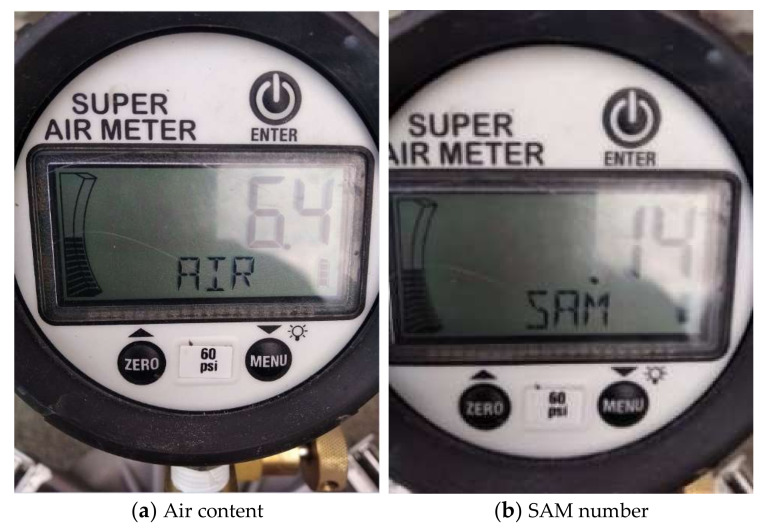
SAM test results (**a**) Air content (**b**) SAM number.

Immediately after mixing (0 min), the slump of fresh mixtures was measured as per ASTM C143 [50]. Meanwhile, the air content was measured by a super air meter (SAM) following AASHTO TP118 [25] as well as a traditional Type B pressure meter following ASTM C231 [3] by different operators. The purpose of the ASTM C231 test was to compare the initial air content. For SAM tests, the SAM number was also acquired after applying successive pressures of 14.5, 30, and 45 psi twice. Figure 1 shows the SAM equipment. Figure 2 presents the air content and SAM number test results. The testing procedure and testing fundamental mechanism of the SAM were briefly introduced as follows. First, the fresh concrete mixtures were placed, consolidated, and leveled in the bottom chamber. Then, the seal and rim between the bottom chamber and lid were cleaned thoroughly. Next, the lid was fixed to the bottom chamber using clamps and the air valve was closed. Both petcocks were then opened and water was injected into one petcock using a rubber syringe until the air-free water emerged from the other petcock. Afterward, the air pump was used to pressurize the top chamber to 14.5 psi as displayed in the digital gauge. After the pressure was stabilized for a few seconds, both petcocks were closed and the lever was pressed to keep the top and bottom chambers in equilibrium. The lever was held for 10 s while tapping the sides of the bottom chamber using a rubber mallet. The equilibrium pressure at this time was used to calculate the air content based on Boyle’s law [24]. Next, the top chamber was pressurized to 30 psi, and the lever was then pressed for 10 s to keep the top and bottom chambers in equilibrium while tapping the sides of the bottom chamber using a rubber mallet. The same step was repeated with a pressure of 45 psi. The equilibrium pressure P_1_ at this time was recorded. Both petcocks were then opened and the water was added through one petcock to the bottom chamber until the air-free water flowed out of the other petcock. After that, the second sequential pressures of 14.5, 30, and 45 psi were also applied and the equilibrium between the top and bottom chambers was reached under each pressure. Finally, the equilibrium pressure P_2_ after finishing the pressure of 45 psi was recorded. The SAM number is calculated as the difference between P_1_ and P_2_ (SAM number = P_2_ − P_1_). Both air content and SAM number were automatically displayed on the digital gauge. More specific details can be found in the AASHTO TP 118 [25]. The calculation of air content was based on Boyle’s law [24], which was the same as the traditional Type B pressure meter. Studies have shown that air voids of different sizes in fresh concrete respond differently to sequential pressures: air voids no larger than 0.2 mm dissolved in solutions under the pressure, while air voids larger than 0.2 mm were just compressed under the pressure and then returned to their original size, so the size and spacing of air voids could be indirectly estimated by applying different pressures [24,26]. Both laboratory and field tests have proved that a SAM number of 0.2 or below could predict a spacing factor of 0.2 mm or below [26,27].

To analyze the air void stability and distribution in fly ash concrete at the fresh state, both air content and SAM number were measured by the SAM at 25 ± 2 min, 50 ± 2 min, and 75 ± 2 min. To simulate the agitation of fresh concrete in the mixer truck during transportation in practice, the concrete mixtures were mixed for 3 min and then rested for 7 min every 10 min. This method was the same as that adopted by Khayat et al. [51] to study the air void stability in self-compacting concrete. During the test, a wet burlap was used to cover the drum mixer to minimize water evaporation. The average of three replicate test results for each mixture was reported.

## 3. Results and Discussion

### 3.1. Fresh Properties

Table 5 presents the fresh properties of eleven fly ash concrete mixtures immediately after mixing. All the slumps were satisfactory as they were between the desired values of 125 mm and 175 mm. The air content obtained by the Type B pressure meter was between 5.8% and 6.7%, while the air content measured by the SAM was between 5.5% and 6.6%. The percentage difference in the air content obtained by the two pieces of equipment was up to 7%. Thus, the SAM resulted in comparable air content measurements to those of a traditional Type B pressure meter. The main reason is that the SAM is a modified version of the traditional Type B pressure meter, and the principle of determining the air content is the same as that of the Type B pressure meter, that is, Boyle’s law [24].

### 3.2. AEA Dosage Demand

The needed AEA dosage to obtain an initial air content of 6 ± 1% for all the fresh fly ash concrete mixtures is listed in Table 6. The workability of concrete mixtures usually affects the effectiveness of the AEA [52]. In this laboratory investigation, all the fly ash concrete mixtures had a comparable slump right after mixing. However, AEA dosage requirements showed relatively large differences, indicating that the various types of fly ash had different adsorption abilities for AEA molecules. The AEA dosage of Class C fly ash (FA1~FA5) concrete ranged from 49 to 90 mL/100 kg cm, while the AEA dosage of Class F fly ash (FA6~FA11) concrete ranged from 56 to 194 mL/100 kg cm.

**Figure 3:**
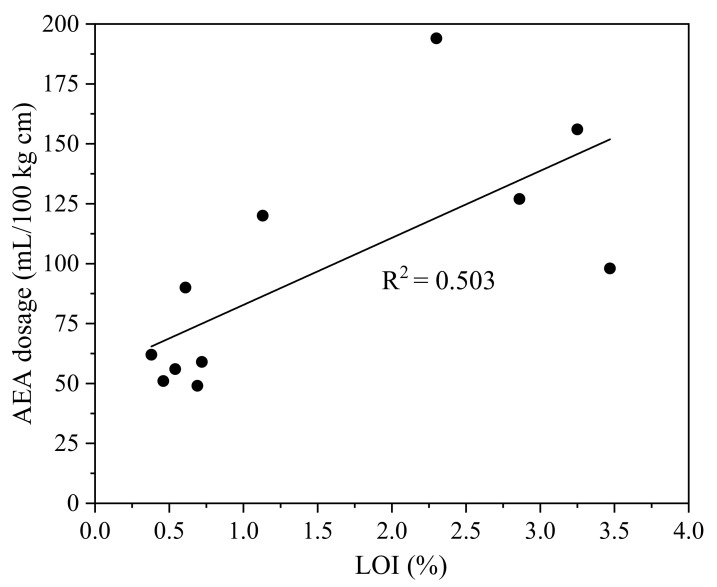
The correlation between the LOI of fly ash and the AEA dosage requirement.

Figure 3 plots the correlation between the LOI of fly ash and the AEA dosage required to obtain an initial air content of 6 ± 1%. Gebler and Klieger [36] reported that the AEA dosage requirement for fly ash concrete increased with the increase in LOI of fly ash. In this study, the correlation coefficient between LOI and AEA demand was 0.503, indicating there was no significant relationship between LOI and AEA demand. Therefore, LOI is not a reliable index to predict the AEA dosage for fly ash concrete. This finding is consistent with the results reported by Schrader [30] and Ley et al. [39]. It has been hypothesized that the residual carbon particles in fly ash reduced the effectiveness of the AEA [41]. The unburnt carbon content is usually evaluated by the LOI, the amount of material lost when burning fly ash at 750 °C. However, several researchers have reported that LOI was unable to accurately determine the unburnt carbon content [53,54,55]. In other words, the residual carbon amount in fly ash is not equal to LOI (carbon amount is less than LOI). The correlation coefficient between the LOI and AEA demand was low likely because the fly ash had other, different characteristics (e.g., specific surface area) that had a greater influence than the LOI or the type of ash. In addition to the amount of carbon, the specific surface area, surface accessibility, and surface chemistry of carbon in fly ash can also contribute to the AEA adsorption [41]. Schrader [30] showed that the correlation coefficient between Brunauer–Emmett–Teller (BET) specific surface area and AEA demand was 0.78. The fine micropores of the carbon surface were not effective for AEA molecules to access [38]. In addition, the surface oxides of carbon from combustion or post-treatment could reduce the AEA adsorption [41]. Moreover, the AEA dosage requirement for fly ash concrete mixtures is associated with the type of AEA [38]. Further study is needed to identify the underlying mechanism and parameters that affect the AEA dosage requirement.

### 3.3. Air Void Stability and Distribution

**Figure 4:**
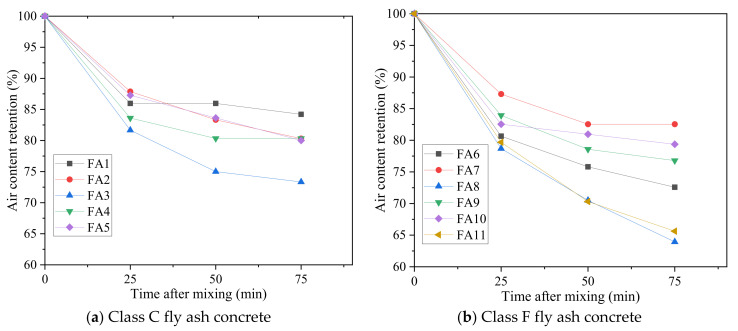
Air content retention of fresh fly ash concrete versus time (**a**) Class C fly ash concrete (**b**) Class F fly ash concrete.

According to SAM tests, the variation in air content and SAM number of fresh fly ash concrete over time after initial mixing was obtained. The air content retention in fresh concrete, defined as the percentage of air content at different times relative to the air content at 0 min, is indicated in Figure 4. The air content of all the fresh concrete showed a decreasing trend with time. The air-entraining admixture (AEA) can lower the surface tension at the interface of air and water, which takes responsibility for generating and stabilizing air voids [56]. However, the adsorption behavior of residual carbon particles in fly ash to AEA molecules was a time-dependent process [38], which caused the decreasing air content. The negative influences of fly ash on the air content retention of fresh mortar or concrete have also been reported by other researchers [36,38,57]. In addition, the spherical shape of fly ash might promote the continuous coalescence of small air bubbles into larger air bubbles, which could induce the air content loss [58]. In addition, the buoyancy force might cause air bubbles to escape from fresh concrete, which could further reduce the air content [59]. It should be noted that SP was used in this study, which sometimes could destabilize air voids in fresh concrete [60,61].

As seen in Figure 4, the most significant reduction in air content primarily occurred within 50 min after initial mixing. As time increased, the adsorption ability of fly ash to AEA molecules gradually weakened. Furthermore, the viscosity of fresh concrete became larger because of the continuous cement hydration and water evaporation; thus, the coalescence, rupture, and escape of air bubbles in fresh concrete were gradually resisted, resulting in the relative stabilization of air content. Once the concrete has hardened, the air bubbles essentially retain their size and shape at the time of setting [52].

Comparing Figure 4a with Figure 4b, it was also observed that most of the Class F fly ash concrete retained their air content worse than Class C fly ash concrete overall. This was likely because Class F fly ash usually has a higher unburnt carbon content than Class C fly ash [34]. In addition, the variation in air content retention was more remarkable for Class F fly ash concrete, ranging from 63.92% to 82.54% at 75 min, compared with that of Class C fly ash concrete, ranging from 73.33% to 84.21% at 75 min.

**Figure 5:**
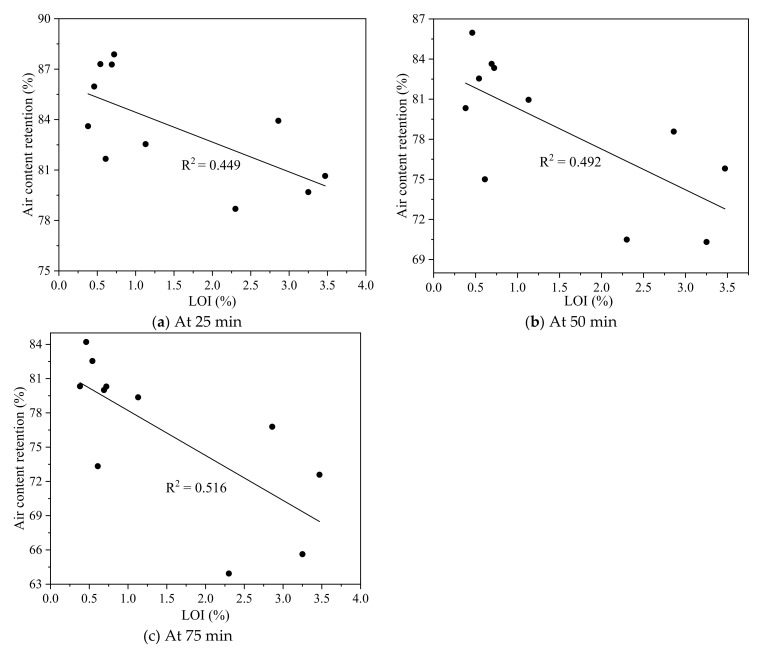
Correlation between air content retention and LOI of fly ash (**a**) At 25 min (**b**) At 50 min (**c**) At 75 min.

Figure 5 presents the relationship between air content retention in fresh fly ash concrete at the sampling times of 25, 50, and 75 min and the LOI of fly ash. The correlation coefficient between the air content retention and the LOI of fly ash at 25, 50, and 75 min was 0.449, 0.492, and 0.516, respectively. This further showed that the LOI of fly ash was not the main factor that affected the air content stability. The low R^2^ presented in Figure 5 was probably because other factors had a larger effect on air content retention, which needs further study in the future. However, a clear tendency about the diminution of air content retention was observed in all the compositions and times, which should be outstanding.

**Figure 6:**
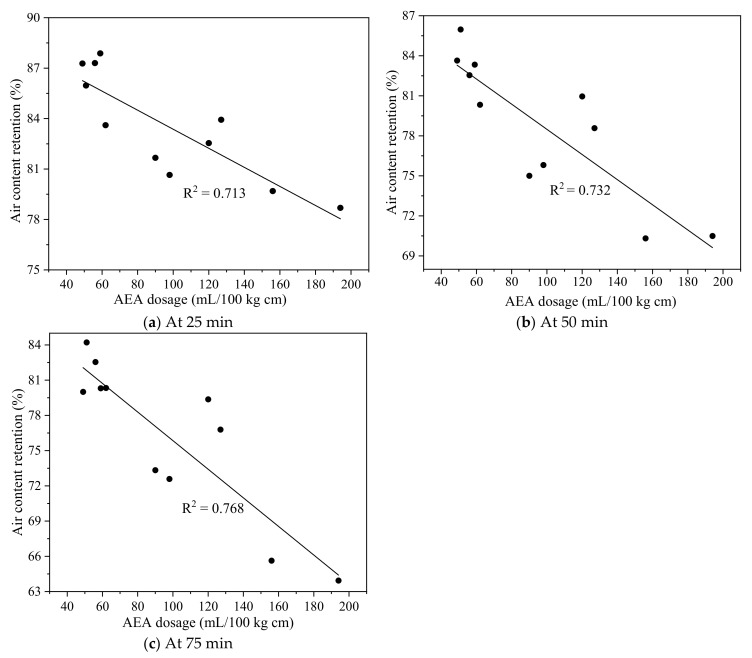
Correlation between air content retention and AEA demand (**a**) At 25 min (**b**) At 50 min (**c**) At 75 min.

The correlation between air content retention of fresh fly ash concrete at 25, 50, and 75 min and AEA demand is plotted in Figure 6. The correlation coefficient between air content retention and AEA demand at the sampling times of 25, 50, and 75 min was 0.713, 0.732, and 0.768, respectively. Compared with LOI, AEA demand exhibited a better correlation with air content retention of fresh concrete. The possible explanation was that fly ash with a higher AEA dosage resulted in a higher adsorption to AEA molecules and, thus, a lower stability of air voids in fresh concrete. Spörel et al. [38] showed that the correlation coefficient between the air content retention after 45 min and AEA demand was 0.93 for fly ash mortar. The lower correlation coefficient in this study might be attributed to the use of SP, which sometimes could induce the destabilization of air voids in fresh concrete [60,61].

**Figure 7:**
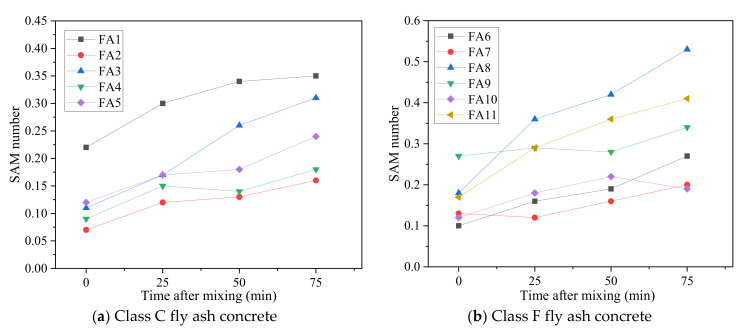
Variation in SAM number of fresh concrete over time (**a**) Class C fly ash concrete (**b**) Class F fly ash concrete.

The variation in the SAM number of fresh concrete over sampling time is shown in Figure 7. A low SAM number indicates a low spacing of air voids in concrete. From a conservative perspective, a SAM number equal to or below 0.2 is desired to achieve a spacing factor no larger than 0.2 mm [26,27]. At 0 min, all the fly ash concrete mixtures had a similar air content of 6 ± 1%, while the SAM number showed a relatively large difference, ranging from 0.07 to 0.27, reflecting the various qualities of the air void system. However, nine out of eleven fly ash concrete mixtures had an SAM number below 0.2, which showed that the air void system was satisfactory right after mixing. Freeze–thaw testing according to ASTM C666 [7] is the most direct method to evaluate the freeze–thaw durability of concrete, but it takes a long time. The American Concrete Institute suggests a spacing factor of no greater than 0.2 mm to make concrete freeze–thaw-durable [31]. However, this test is still conducted on hardened concrete, which is time-consuming. Both lab and field investigations have validated that an SAM number no greater than 0.2 for fresh concrete can be used to predict a spacing factor of 0.2 mm or less, which could ensure that the concrete has enough freeze–thaw resistance [26,27]. Glinicki and Zielinski [62] showed that a satisfactory air void system, i.e., spacing factor smaller than 0.2 mm, was obtained for concrete containing two types of circulating fluidized bed combustion fly ash immediately after mixing. This study indicated that an SAM number below 0.2 was obtained for nine out of eleven fly ash concrete mixtures at 0 min (immediately after the mixing). It was also observed that the SAM number showed an increasing trend with time, and compared with Class C fly ash concrete, the increase in SAM number was more obvious for most of the Class F fly ash concrete mixtures. The spacing of air voids is primarily determined from smaller air voids, i.e., the air voids no larger than 0.3 mm, and, thus, the greater the number of smaller air voids, the smaller the spacing of air voids [63]. As a low SAM number indicated a low spacing of air voids [24,26], the increase in SAM number over time in this study meant the increase in the spacing of air voids, indicating the reduction in the quantity of smaller air voids and the coarsening of the air void structure. This conclusion is in line with the test results of Puthipad et al. [64], who used the Air Void Analyzer (AVA) to investigate the air void stability in fresh self-compacting concrete containing fly ash and concluded that the smaller air voids unified to form larger bubbles during the 120 min interval. Wang et al. [65] showed that adding fly ash beyond a 15% dosage weakened the air void structure and frost durability of hydraulic concrete. The spacing of air voids had a strong correlation with the frost durability of concrete [66]. The increase in SAM number with time for fly ash concrete in this study indicated the increase in the spacing of air voids, which might affect the frost resistance of concrete. Overall, it was concluded that sampling time has a relatively large effect on measuring air content and SAM number for fly ash concrete. The air content and SAM number obtained right after mixing were not necessarily the same as the measured results after 75 min. It could be inferred that the SAM test results obtained in the ready-mix concrete plant might not reflect the air void distribution in fresh fly ash concrete on jobsites before placement. Field tests are essential to validate this finding based on laboratory experiments.

**Figure 8:**
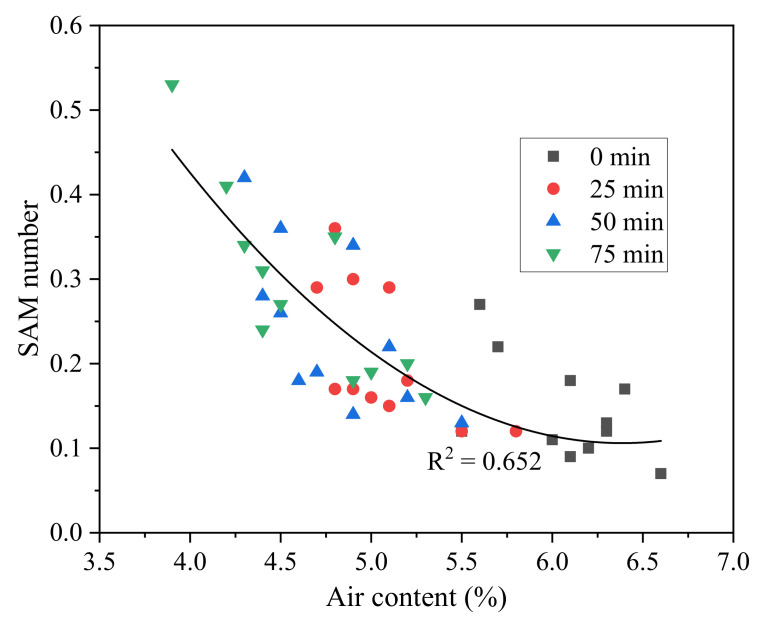
Correlation between SAM number and air content.

Figure 8 plots the correlation between SAM number and air content of fresh concrete at the different sampling times of 25, 50, and 75 min. Previous studies showed that a higher air content could result in a lower spacing factor based on the ASTM C457 test on hardened concrete [27]. This study showed that a higher air content tended to cause a lower SAM number and, thus, a lower spacing of air voids. When the air content was above 6%, all the SAM numbers were below 0.2. The simple explanation was that the increase in air content caused an increased number of air voids and, thus, a decrease in the average distance between air voids, especially when the air content exceeded 6%. Therefore, the advantage of the SAM test is that the spacing of air voids in concrete could be obtained at its fresh state, which facilitates the quality control of air void structures in fresh concrete in real-time. For example, if the measured SAM number (the spacing of air voids) is relatively high, a feasible solution is to increase the air content to achieve a satisfactory air void system.

Overall, the SAM test results of this study showed that there was no special difficulty in producing fly ash concrete with a satisfactory air void system right after mixing, which is consistent with previous studies based on the ASTM C457 test [62]. In addition, the SAM test also showed that the SAM number (spacing of air voids) increased with time, and, thus, the air void system coarsened with time, which is in line with previous AVA test results [63].

## 4. Conclusions

In this study, eleven types of fly ash (five Class C and six class F) were utilized to replace 20% of cement by mass in concrete. Then, eleven fresh fly ash concrete mixtures with an initial air content of 6 ± 1% were produced in the laboratory. The stability and distribution of air voids in fly ash concrete at the fresh state were investigated by a super air meter (SAM). The following main conclusions could be drawn:

(1) There was no significant correlation between the LOI of fly ash and the AEA requirement for the initial air content of 6 ± 1%.
(2) Overall, Class F fly ash concrete had a worse air content retention than Class C fly ash concrete. Compared with LOI, AEA demand had a stronger correlation with air content retention.
(3) Most of the fly ash concrete mixtures had an SAM number below 0.2 right after mixing, indicating that it was feasible to produce fly ash concrete with a high-quality air void system, but the SAM number showed an increasing trend with sampling time, which indicated the coarsening of the air void system. Thus, sampling time had a relatively large effect on measuring air content and SAM number for fly ash concrete. The air content and SAM number obtained right after mixing were not necessarily the same as the measured results after 75 min.
(4) The correlation between air content in fresh concrete and SAM number showed that increasing the air content could reduce the SAM number and, thus, achieve a lower spacing of air voids.

This study presents a laboratory investigation into the air void stability and distribution in fly ash concrete at the fresh state. It is worthwhile to point out that the air void characteristics in concrete are usually affected by many factors, including types of air-entraining admixture and superplasticizer, alkali content in cement, water–cementitious materials ratio, rheological properties (e.g., slump), mixer type, mixing time, pumping, consolidation, placement, and finishing. Therefore, more laboratory tests and field tests on air-entrained fly ash concrete are needed to further verify if similar results will be obtained.

## Figures and Tables

**Table 1:** Chemical compositions and physical properties of cement.

Material	CaO	Al_2_O_3_	SiO_2_	Fe_2_O_3_	K_2_O	Na_2_O	SO_3_	MgO	LOI	Specific Gravity
Cement	63.57	4.35	20.63	3.14	0.48	0.21	2.74	3.2	1.68	3.15

**Table 2:** Classification and physical properties of fly ash.

Materials	FA1	FA2	FA3	FA4	FA5	FA6	FA7	FA8	FA9	FA10	FA11
Class	C	C	C	C	C	F	F	F	F	F	F
Specific gravity	2.71	2.58	2.64	2.75	2.65	2.50	2.61	2.54	2.55	2.57	2.48
Fineness (Retained on No. 325 sieve, %)	22.3	19.6	13.0	20.9	25.4	19.2	26.1	14.7	18.5	11.8	16.4

**Table 3:** Gradation of aggregate.

Sieve (mm)	Fine Aggregate (wt.% Passing)	Sieve (mm)	Coarse Aggregate (wt.% Passing)
9.53	100	50.8	100
4.75	99	38.1	100
2.36	88	25.4	100
1.18	71	19.05	96
0.6	45	12.7	47
0.3	20	9.525	33
0.15	6	4.75	8
0.075	1.2	2.36	3

**Table 4:** Mix proportions of fly ash concrete mixtures.

Cement (kg/m^3^)	Fly Ash (kg/m^3^)	Water (kg/m^3^)	Coarse Aggregate (kg/m^3^)	Fine Aggregate (kg/m^3^)	SP (mL/100 kg cm)	AEA (mL/100 kg cm)
280	70	140	1050	780	90	49~194

**Table 5:** Properties of fresh concrete right after mixing.

Fly Ash Concrete	Slump (mm)	Type B	SAM	
		Air Content (%)	Air Content (%)	SAM Number
FA1	160 ± 3.1	5.9 ± 0.22	5.7 ± 0.16	0.22 ± 0.02
FA2	169 ± 4.6	6.7 ± 0.08	6.6 ± 0.14	0.07 ± 0.01
FA3	158 ± 5.2	6.0 ± 0.14	6.0 ± 0.26	0.11 ± 0.03
FA4	172 ± 2.9	6.4 ± 0.17	6.1 ± 0.29	0.09 ± 0.02
FA5	153 ± 5.6	5.8 ± 0.26	5.5 ± 0.14	0.12 ± 0.03
FA6	155 ± 4.5	6.5 ± 0.16	6.2 ± 0.08	0.10 ± 0.02
FA7	159 ± 6.2	6.2 ± 0.22	6.3 ± 0.25	0.13 ± 0.04
FA8	167 ± 5.9	6.4 ± 0.08	6.1 ± 0.18	0.18 ± 0.04
FA9	163 ± 6.6	5.8 ± 0.16	5.6 ± 0.08	0.27 ± 0.06
FA10	166 ± 4.2	5.9 ± 0.14	6.3 ± 0.24	0.12 ± 0.03
FA11	162 ± 7.3	6.3 ± 0.28	6.4 ± 0.16	0.17 ± 0.05

**Table 6:** The needed AEA dosage to obtain an initial air content of 6 ± 1%.

Fly Ash Concrete	LOI of Fly Ash	AEA Dosage (mL/100 kg cm)
FA1	0.46	51
FA2	0.72	59
FA3	0.61	90
FA4	0.38	62
FA5	0.69	49
FA6	3.47	98
FA7	0.54	56
FA8	2.30	194
FA9	2.86	127
FA10	1.13	120
FA11	3.25	156

## Data Availability

The data presented in this study are available on request from the corresponding author.
